# Quantum Nonlocality

**DOI:** 10.3390/e21050447

**Published:** 2019-04-29

**Authors:** Lev Vaidman

**Affiliations:** Raymond and Beverly Sackler School of Physics and Astronomy, Tel-Aviv University, Tel-Aviv 69978, Israel; vaidman@post.tau.ac.il; Tel.: +972-545908806

**Keywords:** nonlocality, entanglement, quantum

The role of physics is to explain observed phenomena. Explanation in physics began as a causal chain of local actions. The first nonlocal action was Newton’s law of gravity, but Newton himself considered the nonlocal action to be something completely absurd which could not be true—and indeed, gravity today is explained through local action of the gravitational field. It is the quantum theory which made physicists believe that there was nonlocality in Nature. It also led to the acceptance of randomness in Nature, the existence of which is considered as another weakness of science today. In fact, I hope that it is possible to remove randomness and nonlocality from our description of Nature [1]. Accepting the existence of parallel worlds [2] eliminates randomness and avoids action at a distance, but it still does not remove nonlocality. This special issue of Entropy is an attempt to more deeply understand the nonlocality of the quantum theory. I am interested to explore the chances of removing nonlocality from the quantum theory, and such an attempt is the most desirable contribution to this special issue; however, other works presented here which characterize the quantum nonlocality and investigate the role of nonlocality as an explanation of observed phenomena also shed light on this question.

It is important to understand what the meaning of nonlocality is in quantum theory. Quantum theory does not have the strongest and simplest concept of nonlocality, which is the possibility of making an instantaneous observable local change at a distance. However, all single-world interpretations do have actions at a distance. The quantum nonlocality also has an operational meaning for us, local observers, who can live only in a single world. Given entangled particles placed at a distance, a measurement on one of the particles instantaneously changes the quantum state of the other, from a density matrix to a pure state. It is only in the framework of the many-worlds interpretation, considering all worlds together, where the measurement causes no change in the remote particle, and it remains to be described by a density matrix. Another apparent nonlocality aspect is the existence of global topological features, such as the Aharonov-Bohm effect [3]. I believe I succeeded in removing this kind of nonlocality from quantum mechanics [4], but the issue is still controversial [5,6,7,8]. Unfortunately, no contributions clarifying this problem appear in this issue.

It is of interest to analyze nonlocal properties of composite quantum systems, the properties of systems in separate locations [9]. These properties are nonlocal by definition, and the nonlocality of their description does not necessarily tell us that the Nature is nonlocal. It is not surprising that nonlocal properties obey nonlocal dynamical equations. Although unrelated to the question of nonlocality in Nature, it is a useful tool for quantum information which, due to quantum technology revolution, becomes not just the future, but the present of practical applications. See the discussion of this aspect of quantum nonlolcality in this issue and note the recent first experimental realization of measurements of nonlocal variables [10].

For the problem of nonlocality of Nature, the important question is: which of the nonlocal features of composite systems cannot be specified by local measurements of its parts? More precisely, this is the question of nonlocality of a single world, would it be one of the worlds of the many-worlds theory or the only world of one of the single-world interpretations. Even if it does not answer the question of nonlocality of the physical universe incorporating all the worlds, this is the question relevant for harnessing the quantum advantage for tasks which cannot be accomplished classically.

What seems to be an unavoidable aspect of nonlocality of the quantum theory—which is present even in the framework of all worlds together—is entanglement. Measurement on one system does not change the state of the other system in the physical universe, but in each world created by the measurement, the state of the remote system is different. The entanglement, that is, the nonlocal connection between the outcomes of measurements shown to be unremovable using local hidden variables, is the ultimate nonlocality of quantum systems.

Very subjectively—I find the most interesting contribution to be the work by Brassard and Raymond-Robichaud [11], “Parallel Lives: A Local-Realistic Interpretation of ‘Nonlocal’ Boxes”. The work challenges the ultimate question of nonlocality of entanglement. It is part of the ongoing program which was introduced by Deutsch and Hayden [12] to completely eliminate nonlocality from quantum mechanics. The present authors promise to complete it in a future publication. The current paper, instead, provides a wider picture, considering, in a local way, different theories that are currently viewed as nonlocal. The analysis of Popescu Rohrlich (PR) boxes [13], the Einstein–Podolsky–Rosen argument, and Bell’s theorem puts the picture in proper and clear perspective. I am optimistic that Brassard and Raymond-Robichaud will succeed in building their fully local picture as they promise. However, I am also pretty sure that they will have to pay a very high price for removing all aspects of nonlocality by carrying a huge amount of local information in order to reconstruct the consequences of entanglement. Currently, I feel that I will not adopt the “parallel lives” picture, and will stay with the many-worlds interpretation [2], an elegant economical interpretation that has no randomness and action at a distance, but still has nonlocality in the concept of a world. However, I am very curious to see the quantum theory of the parallel lives. The possibility of the construction of a fully local theory, even if it is not economical, is of great importance.

The main test bed for considering nonlocal theories has been the example of PR boxes. It is the topic of the contribution by Rohrlich and Hetzroni [14], “GHZ States as Tripartite PR Boxes: Classical Limit and Retrocausality”. The starting point of this work is Rohrlich’s questioning of his own discovery: can we obtain a classical limit for PR boxes [15]? I am not sure that we have to worry about a classical limit for PR boxes; there is no compelling reason to assume the existence of such a hypothetical construction, as well as the existence of its classical limit. The message of Rohrlich and Hetzroni is that even if the lack of a classical limit for PR boxes represents a conceptual difficulty, there is no difficulty in the case of a quantum-mechanical setup—namely the Greenberger–Horne–Zeilinger setup—which is structurally similar to PR boxes but sufficiently different to have a classical limit. Their paper has also a nice analysis of how retrodiction might solve nonlocality paradoxes.

Retrodiction is also discussed in the contribution by Parks and Spence [16], “Capacity and Entropy of a Retro-Causal Channel Observed in a Twin Mach–Zehnder Interferometer During Measurements of Pre- and Post-Selected Quantum Systems”. The test bed is now a peculiar interferometer considered as a retro-causal channel, analyzed in terms of weak and strong measurements performed on a pre- and post-selected particle. Experimental data collected from an optical experiment performed in 2010 was analyzed. The entropy of this retro-causal structure was considered, making it very relevant for the journal hosting the special issue. The developed formalism is capable of quantitative analysis of other interference experiments.

The level of complexity goes up in the contribution by Bharti, Ray, and Kwek [17], “Non-Classical Correlations in *n*-Cycle Setting”. The compatibility relation among the observables is represented by graphs, where edges indicate compatibility. PR boxes and other nonlocal boxes such as Kochen–Specker–Klyachko boxes are considered for the *n*-cycle case. Non-contextuality is brought up, and extensive analysis of various inequalities characterizing the nonlocality is performed. The work holds the potential to be valuable for the future of quantum computation, as it provides a tight quantitative comparison of efficiency for several tasks of classical methods, quantum methods, and those built on PR boxes.

Another approach for characterizing the nonlocality of quantum theory and some general classes of nonlocal theories (e.g., PR boxes) can be found in the contribution by Carmi and Cohen [18], “On the Significance of the Quantum Mechanical Covariance Matrix”. It also has a direct connection to the journal through the suggestion that the Tsallis entropy quantifies the extent of nonlocality. The key element in this new approach is the connection between nonlocality and a subtle form of uncertainty applicable to general covariance matrices. The most interesting result is that the nonlocality originating from these new characteristics can be measured using feasible weak and strong measurements.

A new approach to harnessing entropic uncertainty relations for investigating quantum nonlocality was presented in the contribution by Costa, Uola, and Gühne [19], “Entropic Steering Criteria: Applications to Bipartite and Tripartite Systems”. Steering may be seen as an action at a distance in one-world interpretations, and thus a robust manifestation of quantum nonlocality. The authors introduce entropic steering criteria, and derive several strong bounds using modest numerical calculations.

A general review of basic techniques for certification of EPR steering was presented by Zhen, Xu, Liu, and Chen [20], “The Einstein–Podolsky–Rosen Steering and Its Certification”. It specified the remaining open problem of how much entanglement is sufficient for EPR steering, and how much EPR steering is sufficient for nonlocality. Solving this problem will advance the realization of nonlocality-based quantum protocols.

Montina and Wolf, in their paper [21] “Discrimination of Non-Local Correlations”, presented a surprisingly efficient algorithm which allowed to answer a very complex problem of characterization of nonlocality using numerical tractable computation. The method shows its validity by successfully reproducing known results, and provides a direction for dealing with difficult, unsolved problems.

Several “loophole-free” Bell-type experiments performed in recent years led to a strong consensus that Nature, or at least the world we live in, has Bell-type nonlocality, but does not have the strong nonlocality of superluminal signalling. Nevertheless, some statistical results of locality testing experiments showed apparently incompatible results. Liang and Zhang, in their paper [22] “Bounding the Plausibility of Physical Theories in a Device-Independent Setting via Hypothesis Testing”, adapted the prediction-based-ratio method (which was originally designed for testing Bell-locality) for testing non-superluminal signaling, the quantum hypothesis, as well as some other natural hypotheses. Their method has provided a unified platform for testing all these different hypotheses at the same time, and is thus a means to evaluate the strength and correctness of various Bell-type experiments.

A paper by Podoshvedov [23], “Efficient Quantum Teleportation of Unknown Qubit Based on DV-CV Interaction Mechanism”, analyzes a novel scheme of qubit teleportation based on continuous variables, arguing that the method is optimal under some realistic constraints. Quantum teleportation is arguably the most spectacular application of quantum nonlocality, as it cannot be explained in the framework of the hidden variables theory.

The question of information transfer in teleportation is, in my view, the key issue in understanding quantum nonlocality [12]. Some light on this question was shed by Cruzeiro and Gisin in their paper [24], “Bell Inequalities with One Bit of Communication”. Their results are based on the development of recent years which showed that the Bell-Type correlations can be simulated by classical means with the help of transmitting a surprisingly small number of bits. They derived a large class of new Bell-type inequalities, and presented a way in which to generate many others.

The formalism of quantum theory allows for the analysis of nonlocal properties which cannot be considered in the classical domain. Classically, a property is either true or false, while in quantum theory, we have the new concept of superposition which has no classical analogue. In the paper [25], “Non-Local Parity Measurements and the Quantum Pigeonhole Effect”, Paraoanu extended the gedanken experiment proposed by Aharonov et al. [26], proposing two constructions of measurement of parity, a manifestly nonlocal variable. This adds a new conceptual twist in the paradox by exposing, in an unexpected way, the tension between quantum physics and local realism.

Quantum nonlocality is not just a peculiar feature which can be harnessed in quantum information tasks—it is also present in many situations. Martínez, Rodríguez, Fierro, Otero, and Aguilar, in their paper [27] “Quantum Nonlocality and Quantum Correlations in the Stern–Gerlach Experiment”, showed the presence of quantum nonlocality in the iconic quantum measurement performed on a single atom.

Quantum nonlocality is an important element for explaining observed quantum effects of organic molecules. Summhammer, Sulyok, and Bernroider analyzed such a situation in their paper [28], “Quantum Dynamics and Non-Local Effects Behind Ion Transition States during Permeation in Membrane Channel Proteins”. The analyzed system is very complex, and some approximations are required, but the observed behaviour was satisfactorily explained only after taking into account quantum nonlocality.

Another work showing the need for quantum nonlocality to explain observed behavior was presented by Iotti and Rossi in [29] “Microscopic Theory of Energy Dissipation and Decoherence in Solid-State Quantum Devices: Need for Nonlocal Scattering Models”. Here, nonlocal generalization of semiclassical (local) scattering models [30] was successful, whereas numeral calculations based on local models failed.

Even if the current special issue does not provide complete answers to all questions about quantum nonlocality, I do see significant progress and am confident that the questions posed here bring us closer to understanding this bizarre feature of quantum mechanics.

## References

[1] Vaidman L. (2014). Quantum theory and determinism. Quantum Stud. Math. Found..

[2] Vaidman L., Zalta E.N. (2018). Many-Worlds Interpretation of Quantum Mechanics. The Stanford Encyclopedia of Philosophy.

[3] Aharonov Y., Bohm D. (1959). Significance of Electromagnetic Potentials in the Quantum Theory. Phys. Rev..

[4] Vaidman L. (2012). Role of potentials in the Aharonov-Bohm effect. Phys. Rev. A.

[5] Aharonov Y., Cohen E., Rohrlich D. (2015). Comment on “Role of potentials in the Aharonov-Bohm effect”. Phys. Rev. A.

[6] Vaidman L. (2015). Reply to “Comment on ‘Role of potentials in the Aharonov-Bohm effect’”. Phys. Rev. A.

[7] Aharonov Y., Cohen E., Rohrlich D. (2016). Nonlocality of the Aharonov-Bohm effect. Phys. Rev. A.

[8] Pearle P., Rizzi A. (2017). Quantum-mechanical inclusion of the source in the Aharonov-Bohm effects. Phys. Rev. A.

[9] Aharonov Y., Albert D.Z., Vaidman L. (1986). Measurement process in relativistic quantum theory. Phys. Rev. D.

[10] Xu X.-Y., Pan W.-W., Wang Q.-Q., Dziewior J., Knips L., Kedem Y., Sun K., Xu J.-S., Han Y.-J., Li C.-F. (2019). Measurements of nonlocal variables and demonstration of the failure of the product rule for a pre- and postselected pair of photons. Phys. Rev. Lett..

[11] Brassard G., Raymond-Robichaud P. (2019). Parallel lives: A local-realistic interpretation of “nonlocal” boxes. Entropy.

[12] Deutsch D., Hayden P. (2000). Information flow in entangled quantum systems. Proc. R. Soc. A Math. Phys. Eng. Sci..

[13] Popescu S., Rohrlich D. (1994). Quantum nonlocality as an axiom. Found. Phys..

[14] Rohrlich D., Hetzroni G. (2018). GHZ States as Tripartite PR Boxes: Classical Limit and Retrocausality. Entropy.

[15] Rohrlich D. (2014). PR-box correlations have no classical limit. Quantum Theory: A Two-Time Success Story.

[16] Parks A., Spence S. (2018). Capacity and Entropy of a Retro-Causal Channel Observed in a Twin Mach-Zehnder Interferometer During Measurements of Pre-and Post-Selected Quantum Systems. Entropy.

[17] Bharti K., Ray M., Kwek L.C. (2019). Non-Classical Correlations in *n*-Cycle Setting. Entropy.

[18] Carmi A., Cohen E. (2018). On the significance of the quantum mechanical covariance matrix. Entropy.

[19] Costa A., Uola R., Gühne O. (2018). Entropic Steering Criteria: Applications to Bipartite and Tripartite Systems. Entropy.

[20] Zhen Y.Z., Xu X.Y., Liu N.L., Chen K. (2019). The Einstein–Podolsky–Rosen Steering and Its Certification. Entropy.

[21] Montina A., Wolf S. (2019). Discrimination of Non-Local Correlations. Entropy.

[22] Liang Y.C., Zhang Y. (2019). Bounding the Plausibility of Physical Theories in a Device-Independent Setting via Hypothesis Testing. Entropy.

[23] Podoshvedov S.A. (2019). Efficient quantum teleportation of unknown qubit based on DV-CV interaction mechanism. Entropy.

[24] Zambrini Cruzeiro E., Gisin N. (2019). Bell inequalities with one bit of communication. Entropy.

[25] Paraoanu G. (2018). Non-Local Parity Measurements and the Quantum Pigeonhole Effect. Entropy.

[26] Aharonov Y., Colombo F., Popescu S., Sabadini I., Struppa D.C., Tollaksen J. (2016). Quantum violation of the pigeonhole principle and the nature of quantum correlations. Proc. Natl. Acad. Sci. USA.

[27] Piceno Martínez A., Benítez Rodríguez E., Mendoza Fierro J., Méndez Otero M., Arévalo Aguilar L. (2018). Quantum Nonlocality and Quantum Correlations in the Stern–Gerlach Experiment. Entropy.

[28] Summhammer J., Sulyok G., Bernroider G. (2018). Quantum dynamics and non-local effects behind ion transition states during permeation in membrane channel proteins. Entropy.

[29] Iotti R., Rossi F. (2018). Microscopic Theory of Energy Dissipation and Decoherence in Solid-State Quantum Devices: Need for Nonlocal Scattering Models. Entropy.

[30] Iotti R.C., Dolcini F., Rossi F. (2017). Wigner-function formalism applied to semiconductor quantum devices: Need for nonlocal scattering models. Phys. Rev. B.

